# Circular RNA Rbms1 inhibited the development of myocardial ischemia reperfusion injury by regulating miR-92a/BCL2L11 signaling pathway

**DOI:** 10.1080/21655979.2022.2025696

**Published:** 2022-01-22

**Authors:** Ling Jin, Yuan Zhang, Yun Jiang, Mingjuan Tan, Caidong Liu

**Affiliations:** Department of Clinical Laboratory, Nanjing First Hospital, Affiliated to Nanjing Medical University, Nanjing, China

**Keywords:** I/R injury, circRbms1, miR-92a, BCL2L11, apoptosis

## Abstract

Acute myocardial infarction (AMI) is characterized by high morbidity and mortality rates. Circular RNAs collectively participate in the initiation and development of AMI. The purpose of this study was to investigate the role of circRbms1 in AMI. Ischemia-reperfusion (I/R) was performed to establish an AMI model. RT-qPCR and Western blotting were performed to detect mRNA and analyze protein expression, respectively. The interaction between miR-92a and circRbms1/BCL2L11 was confirmed by luciferase and RNA pull-down assays. circRbms1 is overexpressed in AMI. However, circRbms1 knockdown alleviated H9c2 cell apoptosis and reduced the release of reactive oxygen species. circRbms1 targeted miR-92a, the downregulation of which alleviated the effects of circRbms1 knockdown and increased oxidative stress and H9c2 cell apoptosis. Moreover, circRbms1 sponged miR-92a to upregulate BCL2L11, which modulated the expression of apoptosis-related genes. circRbms1 participated in myocardial I/R injury by regulating the miR-92a/BCL2L11 signaling pathway, which may provide a new strategy for the treatment of AMI.

## Introduction

Acute myocardial infarction (AMI) is a prevalent heart disease characterized by myocardial necrosis caused by persistent and severe myocardial ischemia. Myocardial ischemia disrupts cellular function, morphology, and metabolism. Patients with AMI are conducive to disability worldwide [1]. Restoring tissue blood perfusion is an effective strategy for AMI treatment [2]. However, emerging myocardial injury, myocardial energy metabolism disorders, and dysfunction-inducing ischemia-reperfusion (I/R) injury offset the clinical results [3,4]. Excessive production of oxygen free radicals (such as nitric oxide), lipid peroxidation, and reactive oxygen species (ROS), is one of the leading causes of I/R injury. Apoptosis plays a vital role in the structural and functional damage of myocardial cells [5]. Unveiling the mechanism of cardiomyocyte death is urgently required.

Circular RNAs (circRNAs) are endogenous RNAs that are reverse-spliced by precursor RNA [6]. Recently, circRNAs have attracted considerable attention due to their crucial roles in cellular functions, such as apoptosis, inflammatory response, and oxidative stress [7]. CircRNAs are also involved in cardiovascular diseases [8]. For instance, circRNA-Las1L inhibits the proliferation and migration of cardiac fibroblasts and promotes cell apoptosis [9]. circRNA-0010729 suppresses injuries to human cardiomyocytes [10]. circRNA ACR has a pro-autophagic role to attenuate myocardial I/R injury in mice by Pink1/FAM65B pathway [11]. However, the role of circRbms1 in AMI remains unclear.

CircRNA contains microRNAs (miRNA) response element, which can bind miRNA as competitive endogenous RNA (ceRNA), inhibit the binding of miRNA to mRNA in cytoplasm and regulate gene expression. This function is called ‘miRNA sponge’. Many reports demonstrated circRNAs modulated the AS progression through functioning as ceRNA. MicroRNAs are small RNAs and many microRNAs were reported to participate in the occurrence and progression of myocardial I/R injury [12]. For instance, miR-206 promotes myocardial hypertrophy and cardiomyocyte survival by mediating YAP signaling [13]. Overexpression of miR-21 in the early AMI stage reduces cell apoptosis and infarct size in the infarct margin area [14]. Additionally, overexpression of miRNA-22 inhibits cardiomyocyte apoptosis, reduces myocardial remodeling, and promotes cardiac function recovery by suppressing microcystin-3 [15]. However, the mechanism of miR-92a in AMI remains unclear.

In this study, we investigated the role of circRbms1 in AMI and its underlying mechanisms. We hypothesized that circRbms1 knockdown alleviated I/R injury via the miR-92a/BCL2L11 axis. These findings provide important insights into the pathology of ischemic heart injury.

## Material and Methods

### Animals

AMI mice model was established according to previous study [16]. Eighteen 8-week-old male C57BL/6 mice were provided by Nanjing Medical University (Nanjing, China). The mice were housed in an SPF environment (room temperature 23°C; humidity 65%; 12 h light/dark cycle), with ad libitum water and food. The hearts of the mice in the model group were exposed by a left thoracotomy. Ligation of the left anterior descending coronary artery (LAD) was then performed. The I/R procedure included ischemia for 1 h and reperfusion for 1, 2, 4, and 8 h. The mice in the sham group underwent a procedure without LAD. Subsequently, the mice were sacrificed and the hearts were collected for the TTC, HE, IHC and TUNEL staining.

### TTC staining

According to a previous study [17]. 2% Evans blue was injected into the femoral vein after LAD ligation. Following injection, the heart tissue was collected immediately and rinsed with frozen normal saline. The heart was frozen at 20°C for 30 min and cut into thin slices with 2 mm thi ck. The sections were incubated with trinitrotoluene at 37°C for 15 min. After being fixed with formaldehyde for 24 h. The sections were imaged. White area indicates the infarcted area, and the red zone indicats the dangerous area (AAR). The infarct size and AAR was calculated by ImageJ (version 1.42) software. Firstly, the infarct size and AAR of each slice was calculated, and then the sum of the infarct size and AAR of each slice multiplied by the thickness (2 mm) was the total size and AAR.

### HE staining

Morphological observation of mice myocardial tissue was conducted HE staining according to a previous study [18]. The left ventricle of the mice was collected and put in 10% formaldehyde. Next, the samples were dehydrated, embedded and cut down into 4 μm slices. After deparaffinage, the sections were stained with hematoxylin and eosin and observed under a light microscope.

### Immunohistochemical (IHC) staining

According to a previous study [19]. The heart slices obtained in HE staining were treated with 3% hydrogen peroxide for 20 min. After that, the slices were incubated with 1% bovine serum albumin and anti-BCL2L11 for 2 h. Then the slices were incubated with horseradish peroxidase (HRP)-conjugated anti-goat IgG for 60 min. Diaminobenzidine (DAB) was selected as chromogen.

### TUNEL assay

TUNEL Apoptosis Assay Kit (Beyotimr, Jiangsu, China) was purchased to measure the apoptosis according to a previous study [20]. The heart tissue was washed and fixed with 4% paraformaldehyde (Beyotime). Then, the heart tissues were washed with phosphate buffer solution (PBS) for twice. Next, PBS containing 0.3% Triton X-100 was added to the heart tissues. Finally, 50 µl TUNEL solution were used for staining, and nuclear cells that labeled positively were considered as apoptotic cells. The positive cells in randomly five views were observed by a fluorescence microscope. The apoptosis rates were presented as positive cells/total cells.

### Cell culture

H9c2 cells were provided by the American Type Culture Collection (VA, USA) and incubated with DMEM supplemented with 10% FBS and 1% penicillin/streptomycin.

The H9c2 cells were exposed to H_2_O_2_ to establish an oxidative stress injury model (200 μM) [21].

### Construction and transfection of plasmid

The oligonucleotide sequence of the expression vector was designed and synthesized by Hanbio (Shanghai, China) according to a previous study [22]. The sequence was as follows: circRbms1 short interference (si) RNA, 5’-TCCAGGCTCAGATGGCAAA-3’; negative control (NC) siRNA, 5’-AGATGAAATTGTGGCTAAA-3.’ The sequence of miR-92a was as follows: 5’-ACAGGCCGGGACAAGTGCAATA-3’. For circRbms1 knockout structures, siRNA oligonucleotides were inserted into the pLVTHM carrier (System Biosciences, CA, USA) located between the MluI and ClaI limiting sites. The BCL2L11 overexpression vector and NC were provided by Shanghai GenePharma (Shanghai, China). The H9c2 cells were seeded in 12-well plates for 24 h. Transfection was performed using the Lipofectin2000 regent (QIAGEN, Hilden, Germany). Chemically modified oligonucleotides (antagomiR) were used to knock down the miRNAs. The antagomir-92a was purchased from RiboBio (Guangzhou, China). We injected antagomiR-92a into the tail vein of rats three consecutive times to silence miR-92a expression *in vivo*.

### Determination of ROS

Intracellular ROS were detected by DCFH-DA staining (Sigma-Aldrich, MO, USA), as previously described [23].

### Flow cytometry

According to a previous study [24], cells were plated in 24-well plates. The H9c2 cells were collected, digested, and centrifuged, and then double-stained with Annexin V-FITC/propidium iodide. Apoptosis was detected by flow cytometry using a BD FACSCalibur (Becton Dickinson, NJ, USA). The gating strategy in flow cytometry was as follows. Firstly, we selected our target group of cells according to FSC and SSC parameters to exclude cell fragments and dead cells. After adjusting fluorescence compensation, we set the gate according to the signal of unstained control cells and single positive stained cells.

### RT-qPCR

RNA was isolated from the cells. Reverse transcription was then performed. RT-qPCR was conducted using SYBR Green mixture (Yisheng, Shanghai). The miRNAs and mRNA were normalized to U6 and GAPDH, respectively. The results were measured using the 2^−ΔΔCt^ method [25].

### RNA pull-down

Biotin-labeled miR-92a probe and NC were provided by Shanghai Sanggong Biotechnology Co., Ltd (China) according to a previous study [26]. The cells were harvested and dissolved in a lysis buffer. The binding reaction was performed using a microliter of magnetic beads. After washing with lysis buffer, the results were analyzed using RT-qPCR.

### Luciferase activity

According to a previous study [27], the binding sites between miR-92a and circRbms1/BCL2L11 were predicted using StarBase 3.0, miRDB, and TargetScan7.2. The wild-type (WT) and mutant type (MUT) of circRbms1 and BCL2L11 were inserted into the psiCHECK vector (Promega, WI, USA). Cells were co-transfected with miR-92a and the 3ʹUTR of WT or MUT of circRbms1/BCL2L11 for 48 h. Luciferase activity was detected by a double luciferase assay (Promega).

### Western blot analysis

Total protein was collected from the H9c2 cells. The protein concentration was calculated using a BCA Kit. Electrophoresis was performed to isolate the proteins, which were subsequently transferred onto polyvinylidene fluoride membranes. The membranes were blocked with 5% skimmed milk and incubated with the primary antibodies (anti-BCL2L11, anti-bid, anti-cyto-c, anti-bcl-2, anti-caspase-3, and anti-β-actin; Boster Biological Technology, Wuhan, China) overnight at 4°C, and then with secondary antibodies. The bands were visualized using a microscope (Bio-Rad, CA, USA).

### Statistical analysis

Data were analyzed using SPSS17.0 (SPSS, IL, USA) and represented as the mean ± SD. One-way ANOVA was applied for analyzing the difference among multiple groups, and Student’s t-test for the difference between two groups. Statistical significance was set at P < 0.05.

## Result

This research demonstrated that circRbms1 and BCL2L11 was over expressed, and miR-92a was decreased in AMI. CircRbms1 knockdown inhibited cardiomyocyte apoptosis and ROS release via regulation of miR-92a/BCL2L11. The circRbms1/miR-92a/BCL2L11 axis may be a potential target for the treatment of I/R injury.

### circRbms1 is overexpressed in myocardial I/R

**Figure 1.**
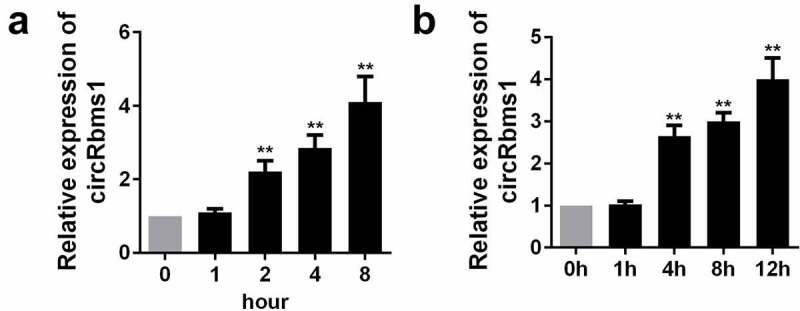
**The expression level of circRbms1 in the I/R mice and H_2_O_2_ treated H9c2 cells**. (a) The level of circRbms1 in the I/R mice was detectedd by RT-PCR. (b) The level of circRbms1 H2O2 treated H9c2 cells was detectedd by RT-PCR. Each experiment was repeated three times. **P < 0.01.

To investigate the role of circRbms1 in AMI *in vivo* and *in vitro* model, we determined its expression levels I/R mice and H_2_O_2_ treated H9c2 cells. The expression of circRbms1 in the I/R mice was increased (Figure 1a). This was consistent with the results of the *in vitro* assays. circRbms1 expression was also upregulated in the H9c2 cells after exposure to H_2_O_2_ (Figure 1b).

### Silencing circRbms1 inhibits H_2_O_2_-induced cardiomyocyte apoptosis and the release of ROS

**Figure 2.**
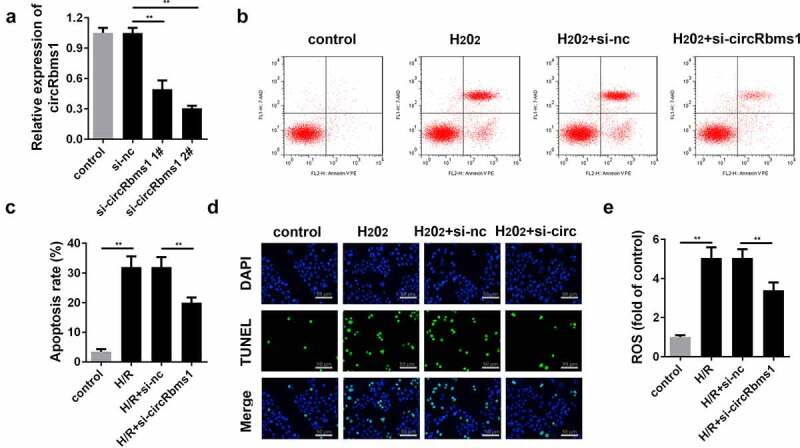
**Silencing circRbms1 can attenuate apoptosis and oxidative stress injury of H9c2 cells induced by H_2_O_2_**. (a) circRbms1 expression was determined by RT-PCR. (b, c) Apoptosis of H9c2 cells was detected using flow cytometry. (d) The apoptosis was detected by TUNEL assays. (e) Reactive oxygen species was assessed. Each experiment was repeated three times. **P < 0.01.

As shown in Figure 2a, the expression of circRbms1 is significantly decreased in cells transfected with circRbms1 knockdown plasmids, which is more potent in the si-circRbms1 2# group. Therefore, si-circRbms1 2# was used in the subsequent experiments. si-circRbms1 knockdown significantly alleviated the increase in apoptosis rates (Figure 2b–d). Moreover, the increase in ROS induced by H_2_O_2_ treatment was significantly attenuated by circRbms1 knockdown (Figure 2e).

### circRbms1 knockdown reduces myocardial I/R injury in vivo via targrting miR-92a

**Figure 3.**
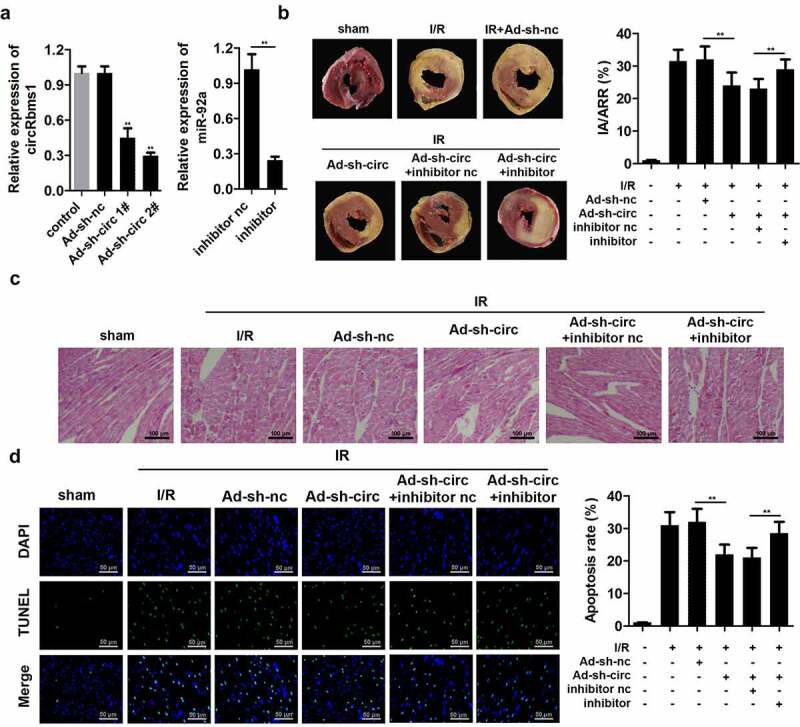
**Silencing circRbms1 inhibits I/R injury in mice**. (a) circRbms1 and miR-92 expression was determined by RT-PCR. (b) The AMI was analyzed using TTC staining. (c) The histopathology examination of myocardial tissues was performed with HE staining. (d) Primary cardiomyocyte apoptosis was evaluated by TUNEL assay. Each experiment was repeated three times. **P < 0.01.

We confirmed the role of circRbms1 in I/R injury using an animal model. circRbms1 expression was remarkably downregulated in mice injected with ad-sh-circRbms1, which was more pronounced in the ad-sh-circRbms1 2# group. Hence, ad-sh-circRbms1 2# was used in the subsequent experiments. Besides, miR-92a inhibitor significantly decreased the miR-92a expression (Figure 3a). Additionally, there was no infarct area in sham group, while the infarct size in the I/R group were significantly increased. CircRbms1 knockdown significantly reduced the infarct size (Figure 3b). Furthermore, HE staining showed that compared to the sham group, clear damage to myocardium could be observed in I/R group. Knockdown of circRbms1 relieved the effects of I/R on myocardial tissue (Figure 3c). Additionally, circRbms1 knockdown also markedly suppressed cardiomyocyte apoptosis (Figure 3d). However, miR-92a knockdown neutralized the sh-circRbms1 effects in the myocardial I/R injury of the mice.

### circRbms1 targets miR-92a

**Figure 4.**
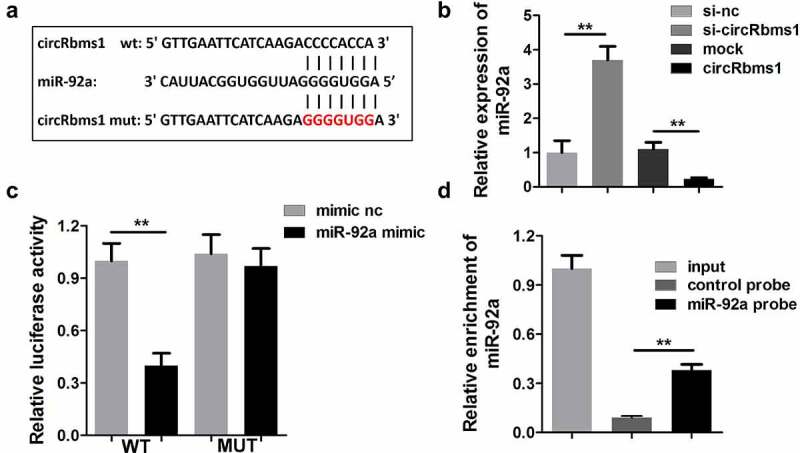
**circRbms1 sponges miR-92a**. (a) The binding sites of circRbms1 and miR-92a. (b) circRbms1 negatively mediates miR-92a expression. The binding sites were confirmed by luciferase report analysis (c) and RNA pull-down assay (d). Each experiment was repeated three times. **P < 0.01.

The possible target miRNAs of circRbms1 were predicted using miRDB and StarBase (Figure 4a). Moreover, miR-92a expression was remarkably downregulated by circRbms1, while, it was upregulated by circRbms1 knockdown (Figure 4b). The binding sites were further confirmed using luciferase activity (Figure 4c), and RNA pull-down assays (Figure 4d).

### miR-92a reverses circRbms1-induced cardiomyocyte apoptosis

**Figure 5.**
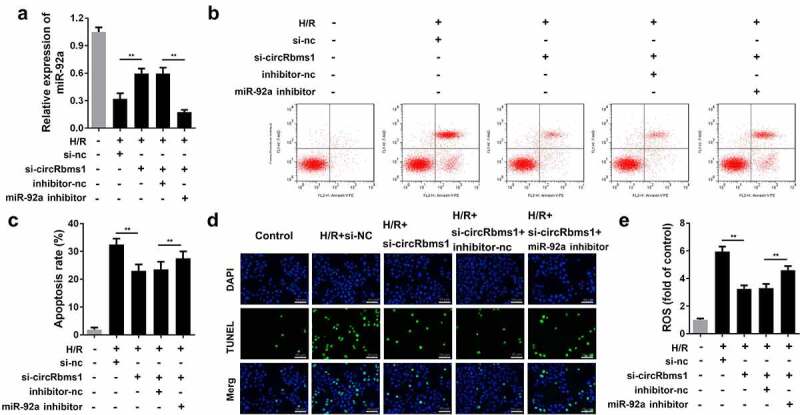
**MiR-92a inhibition reverses the effect of circRbms1 knockdown**. (a) miR92a expression was determined by RT-PCR. (b-d) Apoptosis rates of H9c2 cells were evaluated by flow cytometry and TUNEL staining. (e) The reactive oxygen species in H9c2 cells was detected. Each experiment was repeated three times. **P < 0.01.

circRbms1 acts as a sponge for miR-92a in cardiomyocytes. We performed rescue experiments to study the role of miR-92a in I/R injury. Cardiomyocytes were transfected with si-circRbms1 and an miR-92a-inhibitor. circRbms1 knockdown promoted miR-92a expression and the miR-92a inhibitor notably inhibited this effect (Figure 5a). Rescue experiments indicated that miR-92a downregulation abrogated the effects of circRbms1 knockdown on cardiomyocyte apoptosis (Figure 5b–d). Meanwhile, the miR-92a inhibitor reversed the decrease in ROS that was induced by circRbms1 silencing (Figure 5e).

### miR-92a directly targets BCL2L11 in cardiomyocytes

**Figure 6.**
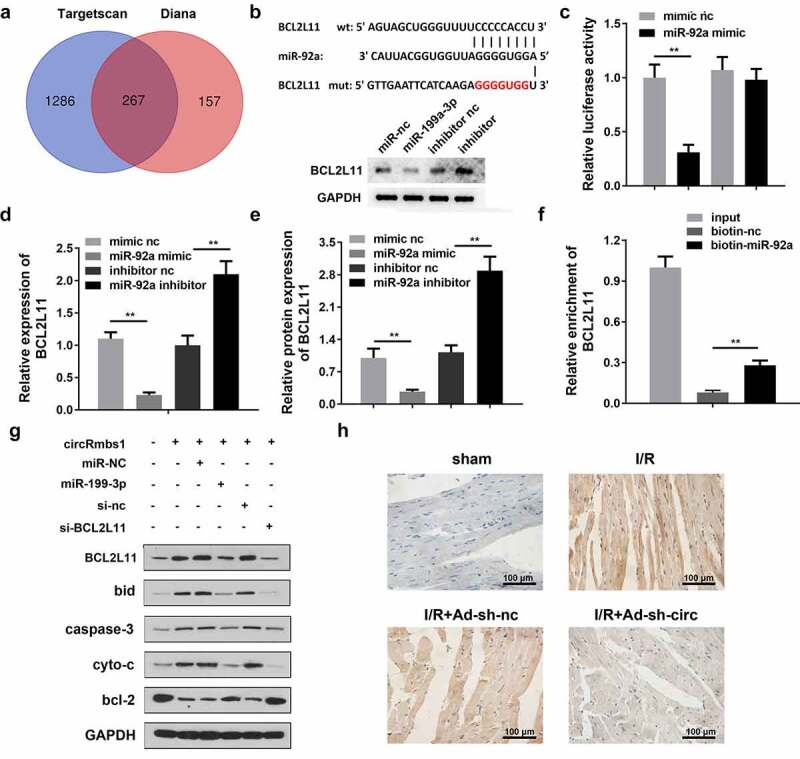
**MiR-92a inhibits cardiomyocyte apoptosis by regulating BCL2L11**. (a) The TargetScan database and Diana database predicted target genes of miR-92a. (b) The binding sites between miR-92a and BCL2L11. (c) The binding sites were confirmed by luciferase report analysis. (d) BCL2L11 expression was determined by RT-PCR. (e) BCL2L11 protein expression was evaluated by Western blot. (f) The binding sites were confirmed by RNA pull-down assay. (g) Protein expression was detected using Western blot. (h) BCL2L11 protein expression was evaluated by IHC staining. Each experiment was repeated three times. **p < 0.01.

TargetScan and Diana databases predicted BCL2L11 as a target of miR-92a (Figure 6a). The target binding regions between miR-92a and BCL2L11 are shown in Figure 6b. miR-92a knockdown improved the protein level of BCL2L11. Moreover, luciferase activity was significantly decreased by co-transfection with the miR-92a mimic and pGL3-3ʹUTR BCL2L11 WT (Figure 6c). RT-qPCR analysis showed that miR-92a markedly negatively regulated BCL2L11 expression (Figure 6d). Additionally, miR-92a overexpression decreased BCL2L11 protein levels, whereas it was promoted by miR-92a knockdown (Figure 6e). An RNA pull-down assay confirmed the interaction between miR-92a and BCL2L11 (figure 6f). As shown in Figure 6g, BCL2L11 knockdown alleviates the effects of miR-92a on the expressions of BCL2L11, caspase-3, bcl-2, bid, and cyto-c. Additionally, the results of IHC staining showed that the BCL2L11 expressions were significantly decreased in heart tissues of the I/R mice after circRbms1 knockdown (Figure 6g)

## Discussion

Myocardial ischemia increases the risk of coronary heart disease and myocardial infarction [28]. Modulation of oxygen consumption, angiogenesis, or cardiomyocyte apoptosis are effective means of treating myocardial ischemia [29]. Therefore, elucidating the underlying molecular mechanisms may provide new strategies for the treatment of AMI. In this study, circRbms1 expression was upregulated in AMI *in vivo* and *in vitro*. Silencing circRbms1 displayed a cardioprotective effect by inhibiting cardiomyocyte apoptosis and the release of ROS. Interestingly, circRbms1 modulated the progression of AMI by regulating the miR-92a/BCL2L11 axis. Thus, circRbms1 may be a potential biomarker for AMI.

circRNAs are key regulators in the development of I/R. Yang et al. revealed that circ-008018 protects against I/R injury by targeting miR-99a [30]. circ-100,338 acts as the cavernous body of miR-200a-3p to promote blood tube formation following myocardial I/R injury [31]. circ-0068566 inhibits the occurrence of myocardial I/R injury by regulating the miR-6322/PARP2 signaling pathway [32]. circRbms1 expression is upregulated during heart failure and is associated with cardiomyocyte apoptosis [33]. In this study, circRbms1 expression was increased in AMI models both *in vivo* and *in vitro*. However, circRbms1 knockdown inhibited cardiomyocyte apoptosis and the release of ROS, which are the key players in the initiation and progression of AMI. Therefore, circRbms1 knockdown may provide protection against AMI by inhibiting cardiomyocyte apoptosis and ROS accumulation. However, the underlying molecular mechanisms remain unclear.

Numerous reports have confirmed that circRNAs may act as molecular sponges to regulate the expression and biological functions of miRNA [34–36]. circRNA-100269 inhibits the development of gastric cancer by targeting miR-630 [37]. Dysregulated circRNA-100290 promotes the aggressiveness of oral cancer through the sponge miR-29 family [38]. In this study, circRbms1 negatively regulated miR-92a expression, which alleviated ischemic injury. Moreover, miR-92a is a critical protective miRNA during cardiac ischemic injury that inhibits inflammatory response and apoptosis [39]. These results suggested that miR-92a may function as a cardioprotective agent, which is consistent with the findings of Song et al. [40]. However, miR-92a downregulation reversed the effects of circRbms1 knockdown and promoted cardiomyocyte apoptosis and ROS release, suggesting that miR-92a may play a beneficial role in AMI and that circRbms1 may participate in the progression of AMI by suppressing miR-92a expression.

Accumulating evidence has revealed that miRNAs interact with their targets to participate in the development of cardiovascular diseases, including AMI [41–43]. miR-130 can target PPAR-γ to aggravate AMI-induced myocardial injury [44]. In this study, BCL2L11 was proved to be a target of miR-92a. BCL2L11 is a key regulator of protein synthesis and cell growth. Activated BCL2L11 promotes myocardial ischemic injury, and its phosphorylation is a crucial step in the protection of cardiomyocytes [45–47]. In this study, BCL2L11 knockdown downregulated the expression of the apoptosis-related genes, caspase-3, bids, and cyto-c, and upregulated the expression of bcl-2. Therefore, circRbms1 modulated BCL2L11 expression to promote the progression of AMI by sponging miR-92a. The circRbms1/miR-92a/BCL2L11 axis may be a potential biomarker for AMI treatment.

## Conclusion

In the present study, circRbms1 was overexpressed in AMI. circRbms1 knockdown inhibited cardiomyocyte apoptosis and ROS release via regulation of miR-92a/BCL2L11. The circRbms1/miR-92a/BCL2L11 axis may be a potential target for the treatment of I/R injury.
